# Protocol for a Sequential Multiple Assignment Randomized Trial to Improve Physical Activity and Diet Quality in Survivors of Cancer

**DOI:** 10.2196/104572

**Published:** 2026-08-13

**Authors:** Eric J Chow, Ethan D Lee, Marie Hershberger, Kari Jenssen, Chongzhi Di, David R Doody, Bridget Hogue, Jason A Mendoza, Tammy M Muller, Jeannette M Schenk, Karen L Syrjala, Yueqi Xu, Jean Yi, Ying-Qi Zhao, Gregory T Armstrong, Marian L Neuhouser, Kevin C Oeffinger

**Affiliations:** 1 Public Health Sciences Division, Fred Hutchinson Cancer Center, PO Box 19024, Seattle, WA, 98109, United States, 1 2066677724, 1 2066675948; 2 Clinical Research Division, Fred Hutchinson Cancer Center, Seattle, WA, United States; 3 Department of Pediatrics, University of Washington School of Medicine, Seattle, WA, United States; 4 Department of Chronic Disease Epidemiology, Yale School of Public Health, New Haven, CT, United States; 5 Department of Biostatistics, University of Washington School of Public Health, Seattle, WA, United States; 6 Department of Epidemiology and Cancer Control, St. Jude Children's Research Hospital, Memphis, TN, United States; 7 Department of Medicine, Duke University School of Medicine, Durham, NC, United States

**Keywords:** survivors of cancer, cardiovascular diseases, lifestyle, physical activity, diet, child

## Abstract

**Background:**

Survivors of childhood cancer have an increased risk of cardiovascular disease (CVD), but that risk may be reduced by healthy lifestyle behaviors.

**Objective:**

Using a sequential multiple assignment randomized trial (SMART) design, this study aims to test different approaches to improve physical activity (PA) and diet quality among long-term survivors of cancer at increased risk of CVD over a 12-month period. The primary study outcomes are sedentary time and Healthy Eating Index (HEI) scores.

**Methods:**

Participants were recruited from the Childhood Cancer Survivor Study cohort, with eligibility defined as current age of 18 to 54 years and at least one of three self-reported criteria: (1) <30 minutes per day of moderate to vigorous PA, (2) HEI-2015 score <60, or (3) BMI ≥25 kg/m^2^. Eligible participants completed baseline surveys, including a food frequency questionnaire, and had PA measured by research-grade accelerometry, and were then randomized to one of three conditions: (1) control, (2) clinician-guided action plan (sessions with the study provider every 2 months), or (3) mobile health–supported goal setting (weekly PA and monthly diet goals based on PA and diet tracker feedback). Following SMART principles, all participants’ PA levels and HEI scores were reassessed after 3 months; participants from the 2 intervention groups who did not achieve ≥20-minute per day reduction in sedentary time or a ≥4-point increase in HEI score were classified as nonresponders and rerandomized to the alternative intervention or a more intensive lifestyle coaching intervention (weekly sessions with a study health coach). Intervention participants then entered a less intensive maintenance phase from months 7 to 12. All participants’ PA levels and HEI scores were reassessed at 12 months. The target sample size was 375 participants, with 95 participants in the control group and 140 participants in each of the initial intervention groups, which will allow the study to detect changes of 0.35 SD (approximately 30 minutes per day) in sedentary time and 0.41 SD (approximately 4 points) in HEI score between the control and intervention groups (combined). Secondary analyses will test whether there are efficacy differences across intervention strategies, and which intervention sequences are associated with the greatest reduction in sedentary time and improvements in diet quality.

**Results:**

From April 2022 to August 2025, the trial completed recruitment and randomized 374 participants out of 2268 approached. The last participant in this ongoing trial is expected to complete all procedures by the end of 2026, after which the final analyses will be conducted and the results will be reported.

**Conclusions:**

Future findings will inform whether any of these intervention strategies (and/or their combinations) can improve PA levels and diet quality in survivors of cancer at increased risk of CVD.

## Introduction

Childhood survivors of cancer now have a 5-year survival rate that exceeds 85%, with over half a million survivors estimated to be living in the United States [1,2]. Early-onset cardiovascular disease (CVD) is now the leading noncancer cause of late mortality among childhood survivors of cancer, with those exposed to cardiotoxic cancer treatments having markedly greater risk [3]. For example, data from the Childhood Cancer Survivor Study (CCSS) cohort found that after a median follow-up of 29 years, CVD accounted for 9% of deaths, trailing only the original cancer (34% of deaths) and new cancers (25% of deaths) [4]. Among CCSS participants, the risk of early ischemic heart disease or cardiomyopathy exceeds 10% by the age of 50 years among those exposed to cardiotoxic cancer treatments [5]. While efforts to minimize cardiotoxic exposures in cancer care have occurred, there remains a critical need to address modifiable risk factors that exist beyond initial treatment and throughout survivorship.

In the general population, low levels of physical activity (PA) and poor diet quality are 2 of the major categories of potentially modifiable lifestyle factors that are also independently associated with increased CVD risk [6]. Specifically, individuals with high levels of sedentary time (more than 9‐10 hours per day) are at the greatest risk [7]. However, even modest reductions in sedentary time could have substantial health benefits [7,8]. Similarly, poor diet quality also contributes independently to increased CVD, with data from large general population cohort studies showing that diet quality is associated with an increased risk of CVD and mortality [6,9,10].

Evidence from the literature, mainly from observational studies, supports the role of lifestyle behaviors, specifically PA and diet, in potentially modifying long-term CVD outcomes among survivors of childhood cancer [4,11,12]. In particular, childhood and adolescent or young adult survivors of cancer often report lower PA levels and poorer diet quality compared with the general population, with barriers including limited knowledge, insufficient provider support, and lack of resources [13-15]. However, survivors have expressed strong interest in interventions aimed at improving lifestyle behaviors, underscoring the need for targeted and accessible strategies [16,17]. To address these gaps, Study of Active LifeStyle Activation (SALSA) was designed to enroll 375 adult survivors of childhood cancer with low PA, poor diet quality, or high BMI, and test different intervention strategies using a sequential multiple assignment randomized trial (SMART) design, with the overall goal of determining overall intervention efficacy and whether specific sequences of interventions can reduce sedentary behavior and improve diet quality in this high-risk population.

## Methods

### Study Design and Theoretical Framework

**Figure 1.**
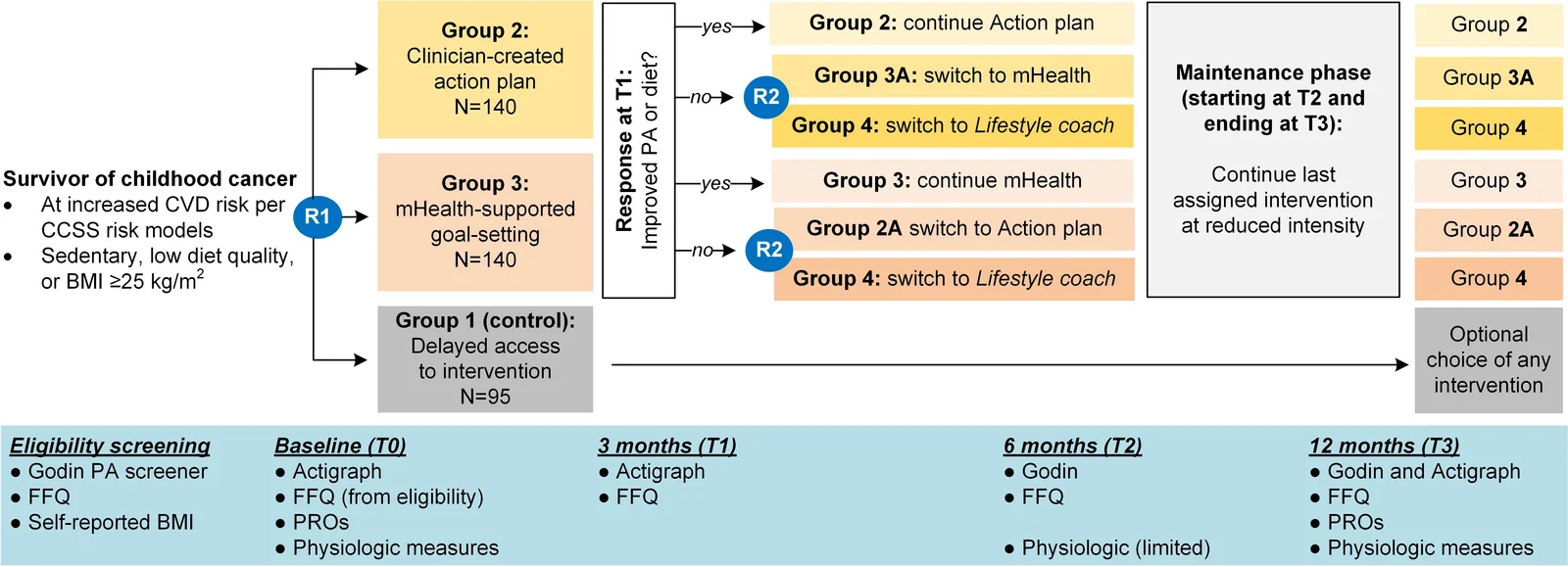
Study schema. CCSS: Childhood Cancer Survivor Study; CVD: cardiovascular disease; FFQ: food frequency questionnaire; mHealth: mobile health; PA: physical activity; PRO: patient-reported outcome.

**Figure 2.**
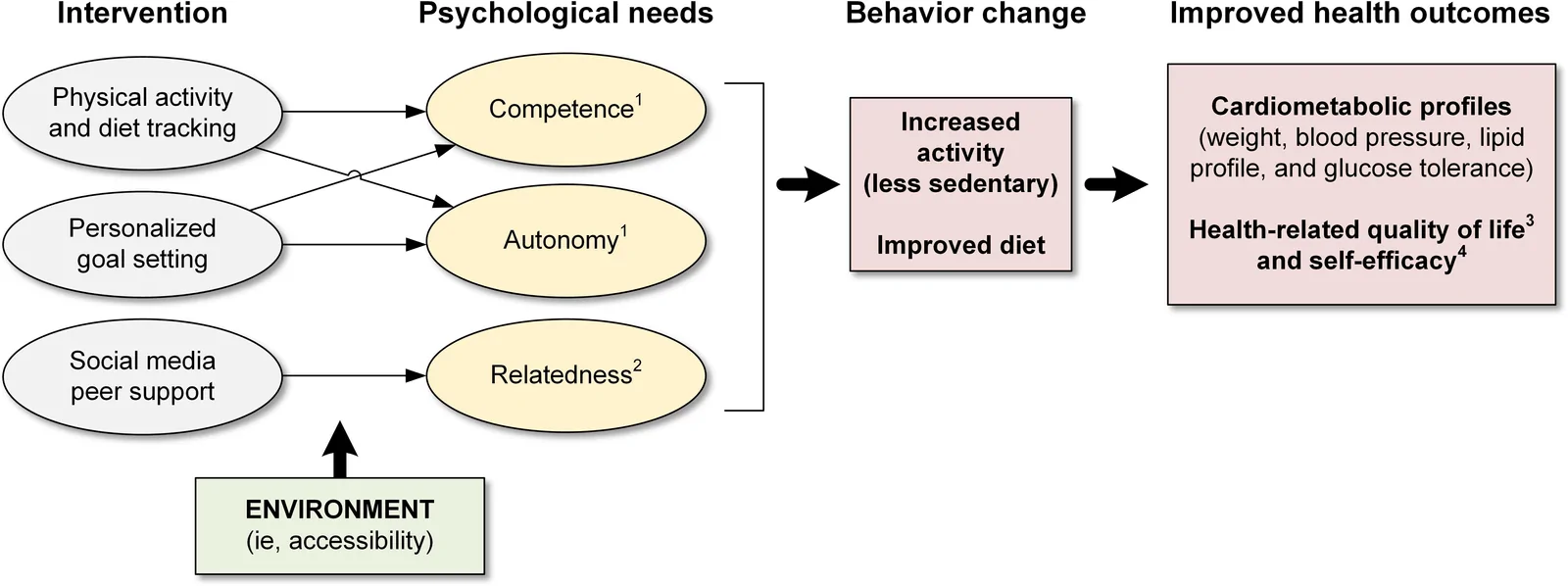
Self-determination theory framework and corresponding patient-reported outcome instruments: (1) self-efficacy toward exercise and diet, diet self-regulation, and behavioral regulation in exercise, (2) ENRICHD (enhancing recovery in coronary heart disease) study, (3) Patient-Reported Outcomes Measurement Information System-10, and (4) per Schwarzer et al [21].

SALSA was based on a SMART design to evaluate the efficacy of different adaptive, remotely delivered behavioral intervention strategies to improve PA and diet quality among survivors of cancer (Figure 1). This design was chosen because it allowed for specific tailoring of interventions based on early participant response, supporting the identification of the optimal sequences for improving lifestyle behaviors among participants [18]. We refer to each such sequence as an embedded adaptive intervention: a prespecified treatment strategy of the form “start with X; if non-responder at 3 months, switch to Y.” SALSA defines 4 embedded adaptive interventions, pairing each of 2 starting interventions with 1 of 2 nonresponder options. The study’s underlying theoretical model for behavior change was based on the self-determination theory (SDT), a well-accepted model supporting PA and dietary change intervention studies [19,20]. Specifically, SDT focuses on increasing individual competence, autonomy, and relatedness (Figure 2).

### Study Population

Participants were recruited from the CCSS, a National Institutes of Health–funded cohort study of over 24,000 5-year survivors diagnosed with cancer before the age of 21 years, between 1970 and 1999, recruited from 31 centers across the United States and Canada, and who have been followed prospectively with periodic surveys [22,23]. For SALSA, eligible CCSS participants had to be aged between 18 and <55 years at the time of study approach, have moderate to high predicted CVD risk based on previously published CCSS-based cardiovascular risk prediction models [24], and meet at least one of three self-reported lifestyle risk criteria: (1) <30 minutes per day of moderate to vigorous PA, (2) Healthy Eating Index (HEI) 2015 score <60, or (3) BMI ≥25 kg/m^2^. These three criteria were chosen given that (1) the US national guidelines recommend ≥150 minutes of moderate PA or ≥75 minutes of vigorous PA (or equivalent combinations of moderate and vigorous PA) per week [25], with the caveat that self-reports tend to overestimate PA relative to accelerometer-based measurement [7]; (2) an optimal HEI score is 100 but the US population mean is 59 [26,27]; and (3) BMI ≥25 kg/m^2^ has been associated with a higher risk of becoming inactive over time, and identified as an independent risk factor for underdiagnosed and undertreated CVD risk factors in childhood survivors of cancer [14,28].

Additional eligibility requirements included fluency in English and internet access. Exclusion criteria included anyone with self-reported ischemic heart disease or cardiomyopathy or heart failure, contraindication to walking or participating in other PA, current pregnancy at time of approach, or being on active cancer treatment. Participants who became pregnant or began new cancer treatment after enrollment were allowed to remain in the study.

### Recruitment

Potentially eligible participants were mailed an approach packet containing an introductory study letter, consent form, a screening questionnaire (self-reported cardiac history, PA levels [29], and current weight and height from which BMI was calculated), and an upfront US $20 incentive in the form of a bank check. Nonresponders received up to 2 additional mailings supplemented by telephone calls and emails over a period of 3 months. If an interested participant did not meet eligibility based on self-reported PA levels and BMI, they were asked to complete the study’s food frequency questionnaire (FFQ) [30], assessing their typical diet over a 1-month time frame, from which the HEI-2015 score was then calculated to determine eligibility.

### Ethical Considerations

All procedures were approved by the institutional review boards at the Fred Hutchinson Cancer Center and St. Jude Children’s Research Hospital, and the study was registered at ClinicalTrials.gov (NCT05075759). All interested participants provided informed consent and had eligibility confirmed by the study team.

### Measurements

At baseline (T0), all eligible and consented participants completed a questionnaire that assessed general health-related quality of life [31], health-related self-efficacy [21], self-efficacy toward exercise and diet [32], self-regulation toward exercise and diet [33,34], the Multidimensional Health Locus of Control [35], and social support [36]. Participants who did not complete the FFQ at screening were also asked to complete it at baseline, and their HEI score was then calculated automatically. Participants’ PA levels and sedentary time over a 7-day period were directly assessed using a research-grade triaxial accelerometry (wGT3x-BT; Ametris), with established algorithms used to detect nonwear time and to define sedentary time (<100 counts per minute) [37,38]. All participants were given Bluetooth-enabled scales and automated oscillometric blood pressure monitors (Withings) to measure their current weight and 3 resting blood pressure values. Measurements were transmitted directly to the study team through the Withings app. Finally, participants were given a dried blood spot (DBS) self-collection kit and asked to return capillary blood for future analysis.

At 3 months (T1), participants were asked to rewear the study accelerometer for 7 days and recomplete the FFQ. At 6 months (T2), participants were asked about their self-reported PA levels [29], repeated the FFQ, and remeasured their weight and blood pressures using their Withings devices. At the final 12-month (T3) time point, all baseline measurements were repeated including the study questionnaire and DBS self-collection.

### Initial Randomization and Intervention Arms

After T0, all participants who completed the questionnaire and FFQ, and had valid accelerometer wear times, were randomized into 1 of 3 groups on a 2:3:3 ratio (control: action plan: mobile health [mHealth]) using a stratified block design (stratified by the study’s eligibility criteria: meeting vs not meeting PA recommendations, HEI score <60 vs ≥60, and BMI <25 vs ≥25 kg/m^2^; Textbox 1). Study staff were blinded to the block size. While the study encouraged participants to complete their self-measured weight, blood pressures, and DBS, randomization and continuing on the study did not require completing those measurements.

Textbox 1. **Overview of control and intervention arm activities.**

- All participants receive at baseline a survivor care plan (SCP)-based telehealth session, delivered by a study nurse practitioner (NP) or certified physician assistant (PA-C)
- Group 1 (control): delayed access to their choice of intervention at the 12-month follow-up (optional)
- Group 2 (action plan): action plan at baseline by NP or PA-C; repeat at 2 months; if ++ response, continue and repeat at 4 and 6 months.
- Group 3 (mobile health [mHealth]): mHealth-supported personalized weekly goal setting and social media peer support; if ++ response at 3 months, continue the intervention through month 6
- Group 4 (lifestyle coach): if poor response to groups 2 or 3 at 3 months, remote sessions with a lifestyle coach every 1 to 2 weeks with advanced dietary and exercise training through month 6
- Maintenance phase (from month 6 to month 12): 
  - Group 2 (action plan): up to 2 additional telehealth sessions
  - Group 3 (action plan): physical activity goals monthly (vs weekly); diet goals remain monthly
  - Group 4 (mHealth): remote sessions once per month

Group 1 (control) received a printed copy of a personalized survivorship care plan (SCP) [39] with recommendations based on the current Children’s Oncology Group guidelines [40]. The SCP content was then reviewed through telehealth with a study clinician (nurse practitioner or physician assistant), generally lasting <30 minutes. Group 1 participants also received complimentary access to a consumer-grade PA tracking watch (Withings Pulse HR with Withings’ app, Withings) and access to a free diet tracking app (Healthwatch360, GB HealthWatch). Group 1 participants received basic instructions on how their devices and apps worked but otherwise received no study-specific feedback on how and when to use these devices and apps.

Group 2 (clinician action plan) participants also received a similar SCP and telehealth review of its content with a study clinician, supplemented with an additional 10 to 15 minutes to codevelop a personalized action plan targeting diet and PA behaviors. The session included structured goal setting, barrier identification, and solution planning based on well-accepted chronic disease self-management models of care [41]. Participants were then offered booster sessions (target ≤15 minutes duration) every 2 months to review and revise the prior action plan as needed, up through 6 months from their first session, to support lifestyle change. Similar to group 1, group 2 participants received the same complimentary digital trackers for PA and diet but did not receive any specific coaching or feedback around their use. To track engagement, the number of sessions participants completed was recorded, and study clinicians also rated participants’ engagement with their action plan after each session on a numeric scale from 0 (no part of the action plan completed and no apparent intention to complete it) to 10 (action plan fully completed).

Group 3 (mHealth) participants received the same SCP and a one-time telehealth session with a study clinician to review their SCP content as group 1. However, group 3 participants also received weekly individualized goals for PA and diet through text or email (per their preference), supported by the study’s complimentary digital trackers (Table 1). PA goals consisted of daily step count targets, while dietary goals included targets for sodium, saturated fat, and added sugar. While these 3 dietary goals only directly accounted for 30% of the HEI-2015 score [26], improvements in these 3 components are associated with improvements in other HEI components and improved overall diet quality [9]. Notably, the available consumer-grade diet trackers available at the time of study implementation were also unable to provide feedback on most of the other HEI components. Participant engagement with these tracking apps was defined as the number of days with ≥500 steps and ≥500 calories recorded [17,42].

**Table 1.** Personalized goal setting categories for group 3 (mobile health).

Goals	Ideal	Borderline	Not ideal
Daily steps	8000‐9999 steps (increase by 10%, up to ≥10,000)^a^	5000‐7999 steps (weekly goal will be to increase daily steps by 10%)	<5000 steps (weekly goal will be to increase daily steps by 500)
Daily added sugars	<10% of total energy (maintain)	10%‐14% (try to reach <10% by next month’s assessment)	≥15% (try to reach 10%‐14% by next month’s assessment and then try to maintain for another month at that level before trying to achieve the ideal range the following month)
Daily saturated fat	<10% of total energy (maintain)	10%‐14% (try to reach <10% by next month’s assessment)	≥15% (try to reach 10%‐14% by next month’s assessment and then try to maintain for another month at that level before trying to achieve the ideal range the following month)
Daily sodium	<2300 mg (maintain)	2300‐3999 mg (try to reduce by 500 mg each month*)*	≥4000 mg (try to reduce by 500 mg each month*)*

a If ≥10,000 steps, maintain and also try to achieve ≥60 active minutes daily.

Group 3 participants were also invited to join a private social media group (private study-specific Facebook group) as a venue for social support and a platform to receive additional information about the health benefits of PA and diet quality, with a focus on the 3 targeted HEI components in the form of a rotating 3-month curriculum (Textbox 2). Specifically, study staff provided links to educational content created by professional groups or organizations (eg, the Centers for Disease Control and Prevention and the American Heart Association), supplemented by articles from the popular press (eg, the National Public Radio and the New York Times, after being reviewed by the study investigators to ensure scientific accuracy). Study staff also sent supportive messaging to participants on the study’s social media site, including weekly recognition of individual and group achievements related to meeting PA and/or dietary goals and study tracker use. Study staff also moderated participant comments and posts, as the social media site is intended to be a forum for participants to discuss and share their experiences with the study’s PA and dietary components. To track engagement, study staff also recorded the number of social media interactions by participants (eg, viewing or liking posts and posting comments).

Due to product discontinuation, the study switched from the Withings Pulse HR to the Withings Scanwatch Light for PA tracking in October 2023. All participants newly enrolled as of this date received the Scanwatch. According to Withings, both products provide identical step count features and interface with the Withings app similarly. Due to the impending discontinuation of Healthwatch360, the study replaced it with Cronometer (Cronometer) for diet tracking in December 2023. Cronometer also offered similar features as Healthwatch360 with the ability to provide feedback on sodium, saturated fat, and added sugar intake, and like Healthwatch, relies on research-grade nutrient databases (eg, the United States Department of Agriculture and the University of Minnesota’s Nutrition Coordinating Center). Further informing these choices, all PA and diet tracking apps used by the study featured researcher portals by which the study team was able to extract participant use data, PA levels, and dietary data directly from the apps.

Textbox 2. **Modular 3-month social media–based curriculum to enhance competence and autonomy toward physical activity (PA) and dietary change.**

**Weekly curriculum**

- Week 0: app introduction or training videos and baseline PA and diet tracking (week 0 can precede week 1a, 1b, or 1c, allowing continuous monthly entry to the intervention. Participants entering at week 1b will end the 3-month block after week 4a. Participants entering at week 1c will end the 3-month block after week 4b)
- Week 1a: group PA and diet averages
- Week 2a: focus on PA
- Week 3a: focus on saturated fats and diet
- Week 4a: review of diet tracking, saturated fats, and PA
- Week 1b: group PA and diet averages
- Week 2b: focus on PA
- Week 3b: focus on sodium and diet
- Week 4b: review of diet tracking, sodium, and PA
- Week 1c: group PA and diet averages
- Week 2c: focus on PA
- Week 3c: focus on added sugars and diet
- Week 4c: review of diet tracking, added sugars, and PA

### Secondary Randomization and Lifestyle Coach Intervention

At 3 months (T1), all participants (including the control group) were assessed for changes in PA levels and/or diet quality. The study defined response a priori if a participant had either a ≥20-minute per day reduction in sedentary time or a ≥4-point increase in HEI-2015 score. These cutoffs were chosen as approximately 50% of participants in an earlier pilot randomized trial would have met at least 1 of these 2 criteria [42]. Participants in the intervention group who did not meet at least 1 of these criteria (ie, nonresponders) were then rerandomized. Nonresponders who were originally assigned to group 2 (clinician action plan) had an equal chance of being rerandomized to the mHealth intervention or a new, more intensive lifestyle health coaching intervention (group 4). Nonresponders originally assigned to group 3 (mHealth) had an equal chance of being rerandomized to the clinician action plan intervention or to group 4. Participants who entered groups 2 and 3 after T1 followed the same intervention schedule as described above for the next 3 months. All participants, including the control group, who did not return evaluable data at T1 despite multiple study attempts were classified as passive withdrawals.

Group 4 participants were scheduled every 1 to 2 weeks over the next 3 months via telehealth sessions with a lifestyle coach with advanced dietary and exercise training. The coaches were tasked with helping participants reduce sedentary time and improve diet quality using a combined cognitive behavioral therapy and motivational interviewing approach [43,44]. Specifically, the coaches counsel within the SDT framework, focusing on realistic goal setting and skill-building, and helping participants identify meaningful motivators related to their personalized health goals. Engagement was tracked similarly to group 2 by recording the number of sessions completed and the coach’s rating after each session.

### Maintenance Phase

To sustain any lifestyle changes, the study included a 6-month lower-intensity maintenance phase where intervention participants had increased responsibility for self-monitoring, goal setting, and other strategies that have been shown to enhance adherence to lifestyle change while still receiving feedback and social support from the study [45]. This phase started 6 months after initial randomization (3 months after secondary randomization). For those assigned to group 2, participants received 2 additional sessions with the study clinician to review their action plans. For those in group 3, PA goals were updated monthly (instead of weekly), while dietary goals remained monthly. Group 3 participants retained access to the study’s Facebook page but no longer received directed, individualized messaging from study staff through that social media platform. For group 4, coaching sessions tapered to once per month.

### Statistical Analysis

All randomized participants will be included in an *intention-to-treat* analysis, focused on detecting differences in sedentary time and HEI-2015 score. Given the SMART design, our primary hypothesis (H1) will test whether there are differences in our 2 primary outcomes between the control group and the intervention groups (combined) using a linear regression model adjusting for any potential confounders that do not appear to be equally distributed across arms, and accounting for baseline sedentary time and HEI values. We will also ensure that the distributions of outcomes and other covariates are not skewed and can transform them if needed. For H1, the estimand is the mean difference in 12-month sedentary time and, separately, the mean difference in 12-month HEI-2015 score, between participants initially assigned to any intervention strategy (groups 2 and 3 combined) vs the control group.

A total sample size of 375 participants, comprising 95 participants in the control arm and 140 participants each in groups 2 and 3, would provide the study with 80% power to detect a change in sedentary time of 0.35 SD (approximately 30 minutes per day) and HEI-2015 score of 0.41 SD (approximately 4 points) between the control arm and the intervention groups (combined). A 30-minute per day reduction in sedentary time among highly sedentary populations has been associated with decreased mortality [7]. Similarly, a 5-point change in HEI score (corresponding to an approximate 1 quintile change) has been associated with a reduction in CVD [9,10]. These power calculations account for dual end points, with a Bonferroni split, where 4% (2-sided) of the alpha is allocated to the sedentary time end point and 1% (2-sided) of the alpha allocated to the HEI end point.

The secondary hypothesis (H2) will test whether there are differences in efficacy between groups 2 and 3. The estimand for H2 is the mean difference between participants initially randomized to the clinician action plan (group 2) and those randomized to the mHealth-supported intervention (group 3), regardless of subsequent adaptation. With 140 participants assigned to each of those groups, the study would have 80% power to detect differences of 0.35 SD and 0.41 SD in sedentary time and HEI score, respectively, accounting for the same 4%/1% Bonferroni split. With these numbers, the study would also be powered to detect (H3) differences of 0.5 SD and 0.6 SD for sedentary time and HEI score, respectively, among those rerandomized to group 4 (lifestyle coach) and those rerandomized to the mHealth and action plan interventions (combined), assuming a 50% response rate at 3 months. For H3, the estimand is the mean difference in 12-month sedentary time and HEI-2015 score among participants classified as nonresponders at 3 months, comparing those rerandomized to the lifestyle coaching intervention (group 4) with those rerandomized to the alternative lower-intensity interventions.

To evaluate the 4 embedded adaptive intervention sequences, we will estimate the mean sedentary time and HEI-2015 score associated with each embedded adaptive intervention using inverse probability weighting to account for the second-stage randomization. Participants will be weighted according to the inverse of their randomization probabilities so that each embedded adaptive intervention represents the target population. Robust variance estimators will be used to obtain valid CIs. These analyses will estimate the mean outcome under each embedded adaptive intervention sequence. The study is not powered for formal pairwise comparisons among the 4 sequences. Other preplanned analyses include examining potential outcome predictors (eg, participant engagement, health-related self-efficacy, and locus of control), mediators (eg, SDT constructs related to competence, autonomy, and relatedness), and moderators (eg, age, sex, and environmental or neighborhood factors based on participant address). We will also explore changes in cardiovascular risk factors (BMI, blood pressures, and DBS-derived laboratory values) over time and investigate their associations with changes in PA profiles and diet quality. Given that the study had to switch its diet tracking app from Healthwatch360 to Cronometer mid-study, we will explore whether there are any engagement differences (ie, number of days with ≥500 calories recorded).

With regard to missing outcomes data, we will carry over a participant’s last observation if they drop out prior to 12 months (T3) to answer H1 and H2. In sensitivity analyses, we can also evaluate the intervention effect size among those with complete 12-month follow-up. Evaluation of H3 and the exploratory analysis examining intervention sequences will be limited to participants who complete the 3-month time point (T1); for those with missing 12-month (T3) data, we will again carry over their last observation. For all these analyses, we will also explore alternative methods to handle missing 12-month outcomes, such as multiple imputation, to evaluate the robustness of the results [46].

## Results

The study began approaching potential participants in April 2022. By the time accrual closed in August 2025, 2268 individuals had been approached. Of these, 577 participants provided informed consent, while 175 interested individuals were excluded after screening and found to be ineligible. Among consenting participants, 374 were randomized, as randomization requires completion of the baseline survey and FFQ plus sufficient accelerometer wear times. Given product changes, the initial 143 participants received the Withings Pulse HR, while the subsequent 231 received the Withings Scanwatch. For dietary tracking, 32 participants randomized to group 3 (mHealth) completed the study using Healthwatch exclusively, while 33 participants started off using Healthwatch before transitioning to Cronometer. All participants newly randomized to mHealth after December 2023 were only given access to Cronometer (n=107). The last study participant of this ongoing trial is expected to complete all study procedures by the end of 2026. Final analyses will then be conducted, and the results are expected to be published thereafter.

## Discussion

SALSA was designed to test different strategies for engaging, promoting, and sustaining lifestyle behavior changes in childhood survivors of cancer at high risk for future CVD over a 1-year period. Much of the preliminary data and impetus for designing SALSA were gained over the course of conducting a preceding randomized trial—CHIIP (Communicating Health Information and Improving coordination with Primary Care; NCT03104543) [47,48]. Although both CHIIP and SALSA recruited high CVD risk survivors from the CCSS, CHIIP focused on helping survivors with hypertension, dyslipidemia, and/or diabetes to improve their cardiometabolic risk factor control, with the randomized group receiving guidance from a study clinician similar to SALSA’s group 2 action plan intervention. The CHIIP intervention featured 1 baseline (approximately 30 minutes) telehealth session to establish the initial goals and action plan, followed by 1 short (approximately 15 minutes) telehealth booster at 4 months to review and revise goals as necessary, with final follow-up occurring 12 months after baseline. Although the clinicians were instructed to prioritize goals that encouraged participants to improve adherence to prescribed medications and to follow up with primary health care providers directly managing these conditions, they were also instructed to provide routine clinical advice about PA and diet, if appropriate. When we reviewed 50 randomly selected CHIIP action plans, we found they contained 48 PA recommendations (ie, nearly all participants) and 61 dietary recommendations (ie, >1 recommendation or participant). We also conducted semistructured qualitative interviews with 20 CHIIP participants, and a consistent theme was a desire for more flexible follow-up options, which participants thought could help increase accountability and improve adherence to goals [49].

As CHIIP was not designed specifically as a PA or diet intervention, SALSA was designed to leverage experiences from CHIIP while also incorporating evidence-based strategies to support lifestyle change. There is a large body of evidence from the general population that counseling on dietary change and PA can be effective, albeit with modest effects [50,51]. The effectiveness of such interventions is increased when they are tailored for a narrower population (eg, those with CVD risk factors) and if they are more intensive and accessible in real time—both strategies were adopted for SALSA [52]. Among survivors of cancer, while there is consistent interest in posttreatment PA and nutritional counseling [53-55], the efficacy of interventions targeting PA and diet quality has been mixed [54,56-58]. However, there is evidence supporting interventions that feature a combined PA and diet focus, including those based on mHealth tools [59,60]. Many of the more effective studies have been intensive with multiple in-person or telephone sessions [61,62]. To enhance efficacy, SALSA also incorporated other evidence-based strategies that enhance adherence to lifestyle change: self-monitoring and goal setting, a focus on self-efficacy, reinforcing positive behavioral change and preventing relapse (of adverse lifestyles), stimulus control, and providing ongoing contact and social support [51,63]. These strategies directly tie into SDT’s focus on individual competence, autonomy, and relatedness.

As SALSA was originally designed in 2020 and funded in 2021, additional data have been published that emphasize the importance of healthy lifestyles in potentially mitigating the long-term impact of adverse cancer treatment exposures on cardiovascular health and overall mortality. For example, updated mortality data from CCSS (n=20,051) found that even after adjusting for cancer treatment exposures, sociodemographic factors, and the presence of conventional cardiometabolic conditions, survivors of childhood cancer who previously reported a healthy lifestyle (no smoking, no risky drinking, and PA >6 metabolic equivalent of task hours per week, and BMI 18.5‐29.9 kg/m^2^) had a 20% lower risk of health-related mortality compared with those with unhealthy lifestyles (ie, having 2 or more adverse lifestyle factors present) [4]. Even those who reported an intermediate lifestyle profile still had a 10% lower risk of mortality compared with the unhealthy lifestyle group.

These findings support the need for prospective randomized trials designed to improve lifestyle patterns in survivors of cancer at high risk of premature CVD. There also remains a preponderance of studies focused on White women with breast cancer, and the evidence base for other survivors of cancer, including survivors of childhood cancer, is much more limited [54,56-59]. SALSA’s focus on upfront intervention strategies that are relatively generalizable (ie, advanced practice provider-based counseling and use of consumer-grade mHealth apps) also increases future dissemination capacity. In summary, lifestyle change represents one of the few available strategies to mitigate cardiovascular risk in childhood survivors of cancer. Significant barriers (eg, time, training, and resources) limit the ability of health care systems to facilitate such change. To fill this void, remote-based, personalized, and easily disseminated multifaceted mHealth-supported interventions may play a transformative role.

## Supplementary material

10.2196/104572 Checklist 1 SPIRIT checklist.

10.2196/104572 Peer Review Report 1 Peer review report by ZCA1 SRB-T (M1) - National Cancer Institute Special Emphasis Panel Research to Reduce Morbidity and Improve Care for Pediatric and AYA Cancer Survivors (National Institutes of Health, USA)

## Abbreviations

CCSS: Childhood Cancer Survivor Study
CHIIP: Communicating Health Information and Improving coordination with Primary Care
CVD: cardiovascular disease
DBS: dried blood spot
FFQ: food frequency questionnaire
HEI: Healthy Eating Index
mHealth: mobile health
PA: physical activity
SALSA: Study of Active Lifestyle Activation
SCP: survivorship care plan
SDT: self-determination theory
SMART: sequential multiple assignment randomized trial
