# Correction: Protocol for a Sequential Multiple Assignment Randomized Trial to Improve Physical Activity and Diet Quality in Survivors of Cancer

**DOI:** 10.2196/111094

**Published:** 2026-09-21

**Authors:** Eric J Chow, Ethan D Lee, Marie Hershberger, Kari Jenssen, Chongzhi Di, David R Doody, Bridget Hogue, Jason A Mendoza, Tammy M Muller, Jeannette M Schenk, Karen L Syrjala, Yueqi Xu, Jean Yi, Ying-Qi Zhao, Gregory T Armstrong, Marian L Neuhouser, Kevin C Oeffinger

**Affiliations:** 1 Public Health Sciences Division, Fred Hutchinson Cancer Center, PO Box 19024, Seattle, WA, 98109, United States, 1 2066677724, 1 2066675948; 2 Clinical Research Division, Fred Hutchinson Cancer Center, Seattle, WA, United States; 3 Department of Pediatrics, University of Washington School of Medicine, Seattle, WA, United States; 4 Department of Chronic Disease Epidemiology, Yale School of Public Health, New Haven, CT, United States; 5 Department of Biostatistics, University of Washington School of Public Health, Seattle, WA, United States; 6 Department of Epidemiology and Cancer Control, St. Jude Children's Research Hospital, Memphis, TN, United States; 7 Department of Medicine, Duke University School of Medicine, Durham, NC, United States

 In “Protocol for a Sequential Multiple Assignment Randomized Trial to Improve Physical Activity and Diet Quality in Survivors of Cancer” [1], the authors noted one omission and two affiliation errors.

Middle initials were absent for several authors. The author list has been revised from the following:

*Eric Chow, Ethan Lee, Marie Hershberger, Kari Jenssen, Chongzhi Di, David Doody, Bridget Hogue, Jason Mendoza, Tammy Muller, Jeannette Schenk, Karen Syrjala, Yueqi Xu, Jean Yi, Ying-Qi Zhao, Gregory Armstrong, Marian Neuhouser, Kevin Oeffinger*

The author list now reads:

*Eric J Chow, Ethan D Lee, Marie Hershberger, Kari Jenssen, Chongzhi Di, David R Doody, Bridget Hogue, Jason A Mendoza, Tammy M Muller, Jeannette M Schenk, Karen L Syrjala, Yueqi Xu, Jean Yi, Ying-Qi Zhao, Gregory T Armstrong, Marian L Neuhouser, Kevin C Oeffinger*

In addition, the listed affiliation for KLS and JY was changed from affiliation 1 to affiliation 2.

The correction will appear in the online version of the paper on the JMIR Publications website together with the publication of this correction notice. Because this was made after submission to PubMed, PubMed Central, and other full-text repositories, the corrected article has also been resubmitted to those repositories.
