# Supplementary material for: Protocol for a Sequential Multiple Assignment Randomized Trial to Improve Physical Activity and Diet Quality in Survivors of Cancer
Source: JMIR Res Protoc. 2026 Aug 13;15:e104572. doi: 10.2196/104572 (PMC13472231; doi:10.2196/104572)
Supplement: Supplementary file 2 [file resprot-v15-e104572-s002.pdf]

SUMMARY STATEMENT
(Privileged Communication)

PROGRAM CONTACT:
Michelle Mollica
240-276-7621
michelle.mollica@nih.gov

Release Date: **03/25/2021**
Revised Date:

Application Number: **1 R01 CA263144-01**

Principal Investigator

CHOW, ERIC JESSEN

Applicant Organization: FRED HUTCHINSON CANCER RESEARCH CENTER

Review Group: **ZCA1 SRB-T (M1)**
National Cancer Institute Special Emphasis Panel
Research to Reduce Morbidity and Improve Care for Pediatric and AYA Cancer Survivors (R01/R21)

Meeting Date: **03/04/2021**
Council: **MAY 2021**
Requested Start: **07/01/2021**
RFA/PA: **CA20-027**
PCC: **MMOR**

Project Title: **SALSA - Study of Active Lifestyle Activation**

SRG Action: **Impact Score:23**
Next Steps: **Visit https://grants.nih.gov/grants/next_steps.htm**
Human Subjects: 30-Human subjects involved - Certified, no SRG concerns
Animal Subjects: 10-No live vertebrate animals involved for competing appl.
Gender: 1A-Both genders, scientifically acceptable
Minority: 1A-Minorities and non-minorities, scientifically acceptable
Age: 3A-No children included, scientifically acceptable

Project Year	Direct Costs Requested	Estimated Total Cost
1	499,978	888,384
2	499,822	888,107
3	499,747	887,974
4	499,630	887,766
5	499,406	887,368
TOTAL	2,498,583	4,439,599

RESUME AND SUMMARY OF DISCUSSION: This study proposes to test a remote sequential multiple assignment randomized trial (SMART) physical activity (PA) and diet intervention in 403 adult survivors of childhood cancer. This study leverages the Childhood Cancer Survivor Study cohort of 24,000 5+ year survivors of childhood cancer, specifically the Chronic Disease working group co-chaired by the PI and Co-I. The application has multiple strengths. The significance is high as children who have had cancer are more than 5 times as likely to suffer from cardiovascular disease as an adult than controls. The investigative team is top-notch and has collaborated on numerous projects. The approach has many strengths, including the SMART design to maximize intervention efficacy and explore the efficacy of different intervention strategies, use of consumer mHealth apps and devices, examination of objective physical activity as well as cardiovascular risk markers using dried blood spots, examination of sustained effects following a maintenance phase, and use of the well-characterized CCSS cohort. Given the team’s previous work and preliminary results, the proposed work is highly feasible. The intensification of the intervention to a lifestyle coach is an evidence-based strategy within the mHeatlh literature that makes sense both based on logic and the available evidence. The level of innovation is moderate to high and the environments for the work are highly supportive and outstanding. Among the many strengths, a few relatively minor concerns are noted. First, more details about eligibility criteria would be helpful. Whether participants are overweight or not at baseline is unclear. Being overweight/obese may or may not be an eligibility criterion if one of the other two criteria is met, and this seems confusing. Second, the age of participants seems excessively broad, and it might be of value to target a more limited range of ages. The investigators suggest that this study focuses on AYA survivors of childhood cancer, but the CCSS cohort was treated between 1970 and 1999 and all of these survivors appear to be eligible for the study. Apparently, the upper limit of age can be as high as 70 (information provided in the clinical section of the application). Third, the intervention uses so many commercial products and each of these products have many features that the team does not control the research ecosystem. As a consequence, it may be difficult to isolate the effect of any specific component of the mHealth intervention, and device or protocol updates may not be in the control of the research team. Fourth, it is also unclear how oversampling of participants of color will be carried out and be successful given the limits of the CCSS cohort. Overall, this application is rated Outstanding.

DESCRIPTION (provided by applicant): Children and adolescents diagnosed with cancer now have, on average, nearly 85% 5-year survival. However, premature cardiovascular (CV) disease has become the leading non-cancer cause of late mortality among childhood cancer survivors. There is a robust body of evidence from the general population, and increasingly, among cancer survivors (even those exposed to cardiotoxic cancer therapies), that greater physical activity (PA) and improved diet quality can reduce future CV-related morbidity. However, while many general population and cancer-specific intervention studies have focused on a single lifestyle factor (e.g., PA or diet alone), given the interplay between PA and dietary factors in influencing CV health, a multi-faceted approach may result in overall better long-term CV health profiles. Research on lifestyle interventions in cancer survivors also has been predominantly conducted in women with breast cancer, and the evidence for survivors of childhood cancer is limited. To accomplish our aims, we will use the largest prospectively followed childhood cancer survivor cohort in the world, the Childhood Cancer Survivor Study (CCSS; n>24,000), to recruit adult-aged participants at increased risk of early CV disease (n=403) for a remotely conducted 12-month randomized controlled trial testing a multi-faceted approach at improving PA and diet quality. Specifically, the study will use a sequential multiple assignment randomized trial (SMART) design, where participants with low PA or poor diet will first be randomized between intervention and control conditions. Intervention participants will be further randomized to receive either clinician-led telehealth sessions focused on risk factor self-management, or weekly mobile health (mHealth) supported individualized PA and dietary goal-setting with social media peer support. The adaptive SMART design will allow further tailoring of the intervention experience based on initial response, which may increase overall intervention efficacy. Participants not initially responsive to their assigned

intervention will be further randomized to receive an alternate intervention. The study will use consumer-grade mHealth applications that track PA and diet, thereby increasing future dissemination capacity. The study's primary analyses will determine the overall intervention efficacy and whether specific intervention strategies and sequence of strategies are associated with optimal outcomes. Secondary analyses will examine potential predictors, mediators, and moderating factors associated with PA and dietary changes over time, as well as changes in participants' cardiometabolic profiles. In summary, lifestyle change represents one of the few available strategies to mitigate CV risk in childhood cancer survivors. Significant barriers (e.g., time, training, resources) limit the ability of healthcare systems to facilitate such change. To fill this void, remote-based, personalized, and easily disseminated multi-faceted mHealth-supported interventions may play a transformative role.

PUBLIC HEALTH RELEVANCE: With improved treatments, the vast majority of children diagnosed with cancer are now cured. However, premature cardiovascular disease has become the leading non-cancer cause of death among childhood cancer survivors. Using an adaptive study design, the proposed trial seeks to determine if various innovative intervention strategies can help childhood cancer survivors improve their physical activity and dietary habits in order to reduce their long-term risk of early cardiovascular disease.

CRITIQUE: The written critiques of individual reviewers are provided in essentially unedited form in this section. Please note that critiques and criteria scores, prepared prior to the review meeting, may not have been revised following discussions at the meeting. The "Resume and Summary of Discussion" section summarizes the final opinions of the review committee.

CRITIQUE 1

Significance: 3
Investigator(s): 1
Innovation: 2
Approach: 3
Environment: 1

Overall Impact*:* Overall, this is a strong application. The investigative team is top-notch and has collaborated on numerous projects. The key players are all executive members of the CCSS project and continue to work together using the large dataset housed at St Jude. The main goal of this project is to examine the efficacy of various intervention strategies to reduce CVD in adult childhood cancer survivors. This goal is clearly important as highlighted in the rationale, children who have had cancer are more than 5 times as likely to suffer from CVD as an adult, than others in the population.
The SMART design is innovative and has the potential to make a major contribution to the field, providing information on various intervention strategies. Testing the potential moderators is also critical within this model.

At its core, this application proposes an obesity intervention, albeit a complex intervention with many interrelated components using the SMART methodological model. As an obesity intervention, BMI is generally an important criterion to consider (although it should never be the sole criteria); however in this application, whether participants are overweight or not at baseline is unclear. In fact, overweight/obese may or may not be an eligibility criterion (if one of the other two criteria are met) and this seems misguided, given the core of the project is on reducing obesity/increasing healthy lifestyles. Similarly, age is not well defined. The investigators suggest that this study focuses on AYA survivors of childhood cancer, but the CCSS cohort were treated between 1970 and 1999 and all of these survivors appear to be eligible for the study. The upper limit of age can be as high as 70 (information provided in the clinical section of the application). Because this is ultimately an obesity/healthy lifestyle intervention,

the limits of such interventions should be considered. Obesity interventions/healthy lifestyle programs are widely available. Because the targets of this project are likely older AYA (and perhaps beyond AYA age group), it is likely that they have engaged in all sorts of 'health' oriented programs in the past. Is this type of information available on the participants? Might this type of information be used as a component of the eligibility criteria or as a potential mediator? Perhaps motivation should be a baseline criterion? Moreover, most of these programs, although important, generally result in weak to at best moderate effects. It is unclear whether the limits of such programs have been considered in developing this project. Again, this is the reason why the age of the participants is so critical – efficacy varies with age, and with mixed aged groups, the findings might end up being even more varied. Can age be limited to the standard AYA age range? Another concern is how oversampling of participants of color will be carried out and be successful given the limits of the CCSS cohort?

1. Significance
Strengths
- Risk of premature CV disease is the leading non-cancer cause of later mortality among childhood cancer survivors
- Using the CCSS cohort to recruit a large number of adult-aged participants at risk for CV and using a SMART design, "sequential multiple assignment randomized trial" procedures are strengths
- Application addresses a knowledge gap; little research has tested tailored interventions targeting adult childhood cancer survivors, and even fewer using SDT as a theoretical guide.

Weaknesses
- In this application, the secondary randomization to alternative strategies is an interesting approach strategy, however, whether a participant does well or not in their first or second condition can presumably be due to other individual factors beyond simply the condition to which they are assigned. The application in fact suggests this in the Innovation section by noting that, "survivors may enter the study with varying levels of motivation and readiness to engage in sustained behavioral change…" Most obesity interventions (presumably many of the adult participants will meet overweight/obesity criteria) are less effective due to numerous factors. If this were not the case, obesity rates would not be as high as they are.
- Perhaps what is missing in the initial assessment is level of motivation as one of the key factors for randomization?
- There is in fact a growing literature on lifestyle interventions that target both PA and dietary behaviors. Some of this literature targets childhood cancer survivors and other chronic illnesses. Overall, most interventions yield a modest effect at best. Whether a tailored mHealth supported intervention based on goal setting fills a gap in the current literature is not necessarily clear. The significance of this application lies in its procedural/statistical approach and not necessarily on the general topic.
- Eligibility criteria includes those factors that are often associated with obesity – higher BMI, poor diet quality and greater sedentary behavior. The general obesity literature – with tons of interventions strategies having been conducted – suggests overall weak to moderate effects for interventions targeting eating and PA behaviors. The intervention used in the current project is based on standard intervention protocols. Although the significance of targeting adult survivors of childhood cancer to attempt to reduce CV morbidity is clear – it is unclear whether this study's intervention protocol includes any discussion of why, as childhood cancer survivors, they should engage in this trial. How will increased engagement be promoted? Often adults who were childhood cancer survivors do not necessarily keep the fact that they had cancer as a child or adolescent at the forefront of their mind. Ultimately, the robustness of the intervention might not yield the effects expected, despite how good the intervention might be – however, this is also an empirical question.

2. Investigator(s).

Strengths

- Professor Chow is a well-regarded scholar in the field, with an extensive track record of funding and research related to the current application
- Dr. Chow is well positioned to carry out this project as he is an executive committee member of the CCSS and has worked closely with his sub-contract PIs (Armstrong and Oeffinger) on the application
- Armstrong and Oeffinger are both top-level scholars in the field and have relevant expertise to contribute to this application.
- The team has a strong history of conducting collaborative cutting-edge research in cancer survivorship and AYA/childhood cancer survivors.
- Chow has organized a team of investigators with relevant expertise to carry out the goals of the application, e.g., experts in behavioral intervention, physical activity and nutrition
- Impressive that the co-I biostatistican, Di, has a current R01 focused on developing novel statistical methods for objectively measuring physical activity data – highly relevant for the current application

Weaknesses

- There are no weaknesses with the team – this is a stellar team, however, there are some related budgetary questions with regard to the team that are listed under the budget category – primarily questioning whether 5% effort is sufficient for several of the co-Is with key roles on the project

3. Innovation

Strengths

- Use of the SMART design is innovative, particularly in cancer-related research, and may yield very important information not necessarily considered in past research.
- Testing the efficacy of different intervention strategies is a strength that the SMART design enables.
- Although not entirely new and innovative, the importance of testing mHealth as a modality to deliver tailored lifestyle interventions for adult childhood cancer survivors is important, particularly when carried out as methodologically rigorous as planned in the current application
- Using the CCSS cohort data with fully remote-based assessments and intervention potentially allows participation from those survivors living in rural and smaller communities where access to survivorship care may be more limited. This is a plus and potentially adds to the innovation of the project.
- The built-in 6 mo lower maintenance phase where participants have increased responsibility for self-monitoring, goal-setting and other strategies is innovative and has the potential to contribute to the intervention literature

Weaknesses

- As noted in the significance section, although important, it is unclear whether testing mHealth as a modality to deliver tailored lifestyle interventions is entirely innovative. There is a growing intervention literature targeting childhood cancer survivors, and for the most part, the effects from these interventions are moderate at best, as are most healthy lifestyle intervention programs, regardless of disease state (or no disease state).
- Although there are clear benefits of using the CCSS cohort, e.g., potential sample size and availability of resources, the age variability of CCSS survivors also adds some complications. The rationale for not using an upper limit in age is unclear.

4. Approach.

Strengths

- Application builds upon “lessons learned” from prior studies conducted by the team – one, investigating a telehealth survivorship care plan and a second, testing a lifestyle intervention.

- SMART design and its potential to test multiple intervention strategies simultaneously is a strength. The study schema as well as the proximal and endpoint measures are appropriate.
- The use of both objective measures of PA via actigraphy and diet via the FFQ/HEI-2015 as well as self-measured assessments of weight, blood pressure and capillary at other points in the project increases the richness of the dataset.
- Collecting data on the neighborhood characteristics in a study such as this is indeed novel
- Utilizing the SDT framework is a strength
- Including the social media venue for social support is a strength of this project
- Considering age, sex and environmental factors are potential moderators in the model is important

Weaknesses (all relatively minor)

- More details about eligibility criteria are needed. Eligibility criteria at baseline includes self-reported BMI. This can of course be problematic, particularly because it seems as if the RCT eligibility requires that only one of several criteria must be met, and BMI is only one of these criteria. Therefore, a participant can potentially participate if they do not eat well or exercise enough, but are not overweight or obese? It is unclear why a BMI corresponding to overweight or obese is not required/a primary eligibility criteria and participants then must meet eligibility for either HEI-2015 and/or MV PA score. In fact, most participants will meet all three of these criteria since over 66% of all adults (now closer to 70%) are overweight or obese and adult childhood cancer survivors are even more likely to meet these criteria.
- How will oversampling of participants of color be successful given the limited number of participants of color who are in the CCSS cohort?
- CCSS cohort encompasses a wide time frame – 1970 to 1999; what will be the mean age of the participants and how will the intervention vary as a function of age? Or at least, what might be the youngest person vs. oldest person participating in this study? What were the ages of those participating in the CHIIP study? Some of this information is provided in the clinical trials section but should also be highlighted in the main body of the application to help with readability. Apparently, the CHIIP study age range was from 20 – 65 and for SALSA will be 21 to 70.
- Is it possible to focus on the AYA portion of this age range, 21 to 39? This would make this application tighter, especially in terms of examining the efficacy of the obesity intervention components. This question about age is critical because obesity interventions, even those that are individually tailored look very different for a 30-year-old than a 70-year-old.
- Is the benefit of conducting this well-designed study with the CCSS cohort as opposed to recruiting from FHRC, as was done with the SmART Heart study, only potential sample size? Being able to target a more restricted age group might ultimately be more beneficial in terms of increasing our understanding of intervention efficacy.
- Using Facebook as a social network is a strength, but as the primary social media outlet for social support raises the issue of age – younger participants may be less likely to use Facebook (however this might not be the case with this population probably being older)?
- Is there a mistake in how participant engagement with mHealth apps for PA is defined? It is stated as number of days with > 500 steps? This goal is obviously very low – but perhaps this was a typing error? If not, more justification is needed.
- The HEI-2015 has not yet been updated with the latest dietary guidelines. If updated during the course of the project, how might these updates affect the study?

5. Environment

Strengths

- Fred Hutchinson Cancer Research Center (FHRC) is one of 51 NCI-designated comprehensive cancer research centers nationwide.
- FHRC and University of Washington have excellent resources available

- St Jude is the largest pediatric oncology center in North America and serves as the coordinating center for the CCSS cohort
- The environment of all the partners (FHRC, St Jude and Duke) on this project are stellar

Weaknesses

- None noted.

Study Timeline

Strengths

- Timeline seems reasonable

Weaknesses

- A More detailed timeline could be presented, given the size and complexity of the project

Protections for Human Subjects

Acceptable Risks and/or Adequate Protections

- Protection of human subjects are adequately addressed

Data and Safety Monitoring Plan (Applicable for Clinical Trials Only):

Acceptable

- o DSMP is acceptable

Inclusion Plans

- Sex/Gender: Distribution justified scientifically
- Race/Ethnicity: Distribution justified scientifically
- Inclusion/Exclusion Based on Age: Distribution justified scientifically
- more information about how exactly greater effort will be expended in attempting to recuit the smaller number available of CCSS participants from minority backgrounds would be useful

Vertebrate Animals

Not Applicable (No Vertebrate Animals)

Biohazards

Not Applicable (No Biohazards)

Select Agents

Not Applicable (No Select Agents)

Resource Sharing Plans

Acceptable

Authentication of Key Biological and/or Chemical Resources

Acceptable

- some blood work will be collected; procedures for collecting DBS (dried blood spots) is well developed and appropriate oversight is included in this application

Budget and Period of Support

- For the FHRC budget:is 5% effort for each co-I sufficient to supervise the development of the intervention? Especially those who will oversee the development of the PA intervention (e.g., Dr Mendoza) and dietary intervention (Prof Neuhouser)
- 5% for Dr Schenk who provides day-to-day assistance with dietary goal-setting questions and FFQ analytic issues seems particularly insufficient

CRITIQUE 2

Significance: 2
Investigator(s): 1
Innovation: 1
Approach: 2
Environment: 1

Overall Impact*:* This study proposes to test a remote sequential multiple assignment randomized trial (SMART) physical activity (PA) and diet intervention in 403 adult survivors of childhood cancer. This study leverages the Childhood Cancer Survivor Study cohort of 24,000 5+ year survivors of childhood cancer, specifically the Chronic Disease working group co-chaired by PI and Co-I. Major strengths of the study include focusing on CCS at increased cardiovascular risk, SMART design to maximize intervention efficacy and explore the efficacy of different intervention strategies, use of consumer mHealth apps and devices, examination of objective physical activity as well as cardiovascular risk markers using dried blood spots, examination of sustained effects following a maintenance phase, and use of the well-characterized CCSS cohort. Given the team's previous work and preliminary results, the proposed work is highly feasible.

1. Significance
Strengths
- Childhood cancer survivors have 5+-fold increased risk of cardiovascular morbidity and mortality and greater burden of hypertension, dyslipidemia, and diabetes
- Reducing sedentary behavior and improving diet quality can reduce CV risk, even among CCS who received cardiotoxic cancer treatments
- Tailored lifestyle interventions for CCS limited
- SMART design allows tailoring of the intervention based on individual responses, to increase intervention efficacy

Weaknesses
- Is the magnitude of change in diet and activity expected to result from the intervention sufficient to significantly reduce cardiovascular risk?

2. Investigator(s).
Strengths
- PI Chow is a pediatric oncologist with expertise in cancer survivorship and multiple relevant publications testing mHealth lifestyle interventions in childhood cancer survivors
- Co-I Oeffinger is an expert in cancer survivorship, co-chairs the CCCS Chronic Disease Working Group with PI Chow
- Co-I Di is a biostatistician with expertise in methods to analyze physical activity data and who has collaborated with this team
- Co-I Mendoza is a pediatrician with expertise in mHealth behavioral interventions to eliminate disparities in nutrition and PA in AYACS
- Co-I Neuhouser is an expert in dietary assessment and intervention
- Co-I Schenk is a nutritionist and epidemiologist with expertise in lifestyle interventions and mHealth for dietary tracking
- Co-I Syrjala is an expert in biobehavioral oncology, including AYACS survivorship, digital interventions, behavioral RCTs, and SMART designs
- Co-I Yi has expertise in technology-based interventions for cancer survivors
- Co-I Zhao is a biostatistician with expertise in design and analysis of SMART trials
- Consortium PI Armstrong is PI of the CCCS and has expertise in long-term outcomes in pediatric cancer survivors
- Record of prior collaboration, including a relevant 2020 publication involving Chow, Doody, Di, Schenk, Syrjala, Neuhouser, Mendoza

- Table specifying role for each investigator

Weaknesses
- None noted.

3. Innovation
Strengths
- Uses mHealth to deliver the intervention
- SMART design to tailor the intervention based on initial response
- Leverages the large well-characterized CCSS cohort
- Fully remote, with Bluetooth devices for blood pressure and weight assessment and dried blood spot for CV risk analytes

Weaknesses
- None noted.

4. Approach.
Strengths
- Primary outcomes are accelerometry-measured sedentary time and improved diet quality (HEI-2015)
- Secondary outcomes include cardiovascular risk markers, including dried blood spot cholesterol, CRP, glucose, etc as well as weight and blood pressure from WiFi devices
- Enrolling survivors predicted to have moderate to high risk of future CV disease (based on sedentary behavior, diet quality, or overweight/obesity)
- Maintenance phase to examine whether lifestyle changes are sustained
- Intervention is grounded in self-determination theory, with components based on chronic disease self-management models, cognitive-behavioral therapy, and motivational interviewing
- Arm #1 intervention is generalizable to clinical practice
- Reasonable accrual goal given number of eligible participants in CCSS or prior successes recruiting from this cohort
- Oversampling individuals from minority groups to recruit a diverse sample
- Preliminary data supports feasibility of the proposed approach, and proposed work aims to improve on earlier studies based on participant feedback/results
- Control condition will have access to health trackers and delayed access to intervention
- Uses consumer activity and diet tracking apps, with backup plans if technology changes
- Assessment of neighborhood factors as moderating variables

Weaknesses
- Peer support through Facebook – although educational content will be made available to those who elect not to join this group, 30% or more may miss out on peer support and discussion
- Poor adherence to diet tracking in prior work but limited plans to improve

5. Environment
Strengths
- FHCRC, UW, SJCRH, and Duke offer excellent resources for the proposed research
- Access to the CCCS in a noteworthy strength of the study

Weaknesses
- None noted.

Study Timeline
Strengths
- Reasonable timeline for accrual

Weaknesses
- None noted.

Protections for Human Subjects
Acceptable Risks and/or Adequate Protections
Data and Safety Monitoring Plan (Applicable for Clinical Trials Only):
Acceptable

Inclusion Plans
- Sex/Gender: Distribution justified scientifically
- Race/Ethnicity: Distribution justified scientifically
- For NIH-Defined Phase III trials, Plans for valid design and analysis: Scientifically acceptable
- Inclusion/Exclusion Based on Age: Distribution justified scientifically

Vertebrate Animals
Not Applicable (No Vertebrate Animals)

Biohazards
Not Applicable (No Biohazards)

Select Agents
Not Applicable (No Select Agents)

Resource Sharing Plans
Acceptable

Authentication of Key Biological and/or Chemical Resources
Acceptable

Budget and Period of Support
Recommend as Requested

CRITIQUE 3

Significance: 2
Investigator(s): 1
Innovation: 2
Approach: 2
Environment: 1

Overall Impact*:* The proposed SMART trial addresses an important problem of cardiovascular risk brought on by poor diet and high sedentary behavior in cancer survivorship. This is a high impact application as it uses appropriate behavioral and biological endpoints, uses an appropriate theoretical model, and will use a sophisticated research methodology to optimize care for intervention participants. The methodology will allow comparisons of three initial intervention levels (which is itself a strength) and then will be able to determine whether non-responders experience improved outcomes with a switch to another intervention modality. Importantly, the intensification of the intervention to a lifestyle coach is an evidence-based strategy within the mHeatlh literature that makes sense both based on logic and the available evidence. There is one major weakness in the design. Specifically, the intervention uses so many commercial products and each of these products have so many features that the team does not control the research ecosystem. As a consequence, it may be difficult to isolate the effect of any specific component of the mHealth intervention and in a worst case scenario, device or protocol updates will not be in the control of the research team.

1. Significance
Strengths
- Cardiovascular disease is an important contributor to mortality and a risk factor for AYAs who have survived cancer.
- Physical activity and diet are modifiable health behaviors consistent with the NCI strategic plan that can reduce the burden of cardiovascular disease and these linkages are made explicit in the proposal.
- The ability to leverage the CCSS cohort is a major strength and will link the trial to a number of other variables.
- The

Weaknesses
- None noted.

2. Investigator(s)
Strengths
- The team is excellent with the requisite experience to lead the work.
- Dr. Chow has directed multiple high impact studies that inform the practical and conceptual makeup of this project.

Weaknesses
- Prior work on mHealth approaches to health behavior in survivorship is not fully integrated in the significance section.

3. Innovation
Strengths
- The use of the SMART design is innovative for this problem and is one of the fastest paths to optimization. It is a good design choice in addition to the innovation.
- The reach of the CCSS for recruitment presents an important innovation to scale up the recruitment pool and enhance equity.

Weaknesses
- A mHealth intervention for physical activity and diet is not in and of itself innovative.

4. Approach
Strengths
- The application is very well supported by pilot work in mHealth applications (Smart Heart and a stand alone physical activity intervention).
- The RCT design with rerandomization is a strength and adds rigor and optimization.
- Blocking for the study appears appropriate as to the inclusion criteria.
- The control condition is appropriate and concerns about reactivity to the fitbit are allayed by the length of the protocol.
- Intervention decisions (e.g., personalized goals) are directly informed by prior pilot work and represent empirical choices. This is uncommon in such trials and is a major strength.
- Both PA and diet are measured via rigorous assessments that are appropriate for each outcome.
- Biological outcomes are proposed that are quite rigorous for remote collection.

Weaknesses
- The incentives offered in the project may be smaller than needed for follow-up timepoints.
- A large number of commercial products are used and each of these products have so many features that the team does not control the research ecosystem.

5. Environment
Strengths

- The environment is excellent, and the investigators have achieved considerable success using the available resources.

Weaknesses

- None noted.

Study Timeline

Strengths

- The timeline is detailed and appropriate for the aims.

Weaknesses

- None noted.

Protections for Human Subjects

Acceptable Risks and/or Adequate Protections

- The human subjects section is detailed and appropriate.

Data and Safety Monitoring Plan (Applicable for Clinical Trials Only):

Acceptable

- o DSMP is appropriate.

Inclusion Plans

- Sex/Gender: Distribution justified scientifically
- Race/Ethnicity: Distribution justified scientifically
- Inclusion/Exclusion Based on Age: Distribution justified scientifically
- Rates of cancer are used to justify expected recruitment.

Vertebrate Animals

Not Applicable (No Vertebrate Animals)

Biohazards

Not Applicable (No Biohazards)

Select Agents

Not Applicable (No Select Agents)

Resource Sharing Plans

Acceptable

Authentication of Key Biological and/or Chemical Resources

Not Applicable (No Relevant Resources)

Budget and Period of Support

Recommend as Requested

THE FOLLOWING SECTIONS WERE PREPARED BY THE SCIENTIFIC REVIEW OFFICER TO SUMMARIZE THE OUTCOME OF DISCUSSIONS OF THE REVIEW COMMITTEE, OR REVIEWERS' WRITTEN CRITIQUES, ON THE FOLLOWING ISSUES:

PROTECTION OF HUMAN SUBJECTS: ACCEPTABLE

INCLUSION OF WOMEN PLAN: ACCEPTABLE

INCLUSION OF MINORITIES PLAN: ACCEPTABLE

INCLUSION ACROSS THE LIFESPAN: ACCEPTABLE

COMMITTEE BUDGET RECOMMENDATIONS: The budget was recommended as requested.

Footnotes for 1 R01 CA263144-01; PI Name: Chow, Eric Jessen

NIH has modified its policy regarding the receipt of resubmissions (amended applications).See Guide Notice NOT-OD-18-197 at https://grants.nih.gov/grants/guide/notice-files/NOT-OD-18-197.html. The impact/priority score is calculated after discussion of an application by averaging the overall scores (1-9) given by all voting reviewers on the committee and multiplying by 10. The criterion scores are submitted prior to the meeting by the individual reviewers assigned to an application, and are not discussed specifically at the review meeting or calculated into the overall impact score. Some applications also receive a percentile ranking. For details on the review process, see http://grants.nih.gov/grants/peer_review_process.htm#scoring.

MEETING ROSTER

National Cancer Institute Special Emphasis Panel
NATIONAL CANCER INSTITUTE
Research to Reduce Morbidity and Improve Care for Pediatric and AYA Cancer Survivors (R01/R21)

ZCA1 SRB-T (M1)
03/04/2021 - 03/05/2021

Notice of NIH Policy to All Applicants: Meeting rosters are provided for information purposes only. Applicant investigators and institutional officials must not communicate directly with study section members about an application before or after the review. Failure to observe this policy will create a serious breach of integrity in the peer review process, and may lead to actions outlined in NOT-OD-14-073 at https://grants.nih.gov/grants/guide/notice-files/NOT-OD-14-073.html and NOT-OD-15-106 at https://grants.nih.gov/grants/guide/notice-files/NOT-OD-15-106.html, including removal of the application from immediate review.

CHAIRPERSON(S)

FRIEDMAN, DEBRA L, MD
PROFESSOR
DIVISION OF PEDIATRIC ONCOLOGY AND HEMATOLOGY
E. BRONSON INGRAM CHAIR AND DIRECTOR
VANDERBILT UNIVERSITY
NASHVILLE, TN 37232

HENDERSON, TARA OLIVE, MD, MPH
PROFESSOR
DEPARTMENT OF PEDIATRIC HEMATOLOGY, ONCOLOGY
AND STEM CELL TRANSPLANTATION
THE UNIVERSITY OF CHICAGO MEDICINE
CHICAGO, IL 60637

MEMBERS

AUNE, GREGORY J., MD, PHD
ASSISTANT PROFESSOR
DEPARTMENT OF PEDIATRICS HEMATOLOGY/ONCOLOGY
UNIVERSITY OF TEXAS HEALTH SCIENCE CENTER
SAN ANTONIO, TX 78207

BARAC, ANA, MD, PHD
DIRECTOR CARDIO-ONCOLOGY
DEPARTMENT OF CARDIO-ONCOLOGY PROGRAM
CARDIAC REHABILITATION PROGRAM
MEDSTAR WASHINGTON
GEORGETOWN UNIVERSITY
WASHINGTON, DC 20057

BUCHBINDER, DAVID KYLE, MD
PROFESSOR
DEPARTMENT OF HEMATOLOGY AND PEDIATRICS
UNIVERSITY OF CALIFORNIA AT IRVINE
IRVINE, CA 92697

COLLINS, LINDA M, PHD
PROFESSOR
DEPARTMENT OF SOCIAL AND BEHAVIORAL SCIENCES
AND BIOSTATISTICS
CENTER FOR DRUG USE AND HIV RESEARCH
NEW YORK UNIVERSITY
NEW YORK CITY, NY 10003

CUSHING, CHRISTOPHER C, PHD
ASSISTANT PROFESSOR/ASSISTANT SCIENTIST
DEPARTMENT OF CLINICAL CHILD
PSYCHOLOGY PROGRAM
UNIVERSITY OF KANSAS
LAWRENCE, KS 66045

DOCHERTY, SHARRON L, PHD, FAAN
ASSOCIATE PROFESSOR AND DIRECTOR
DEPARTMENT FOR THE CENTER FOR COGNITIVE
AND AFFECTIVE SYMPTOM SCIENCE
DUKE UNIVERSITY SCHOOL OF NURSING
DURHAM, NC 27710

DORRIS, KATHLEEN, MD
ASSOCIATE PROFESSOR
DEPARTMENT OF PEDIATRICS
CHILDRENS HOSPITAL COLORADO
UNIVERSITY OF COLORADO
AURORA, CO 80045

FEDERMAN, NOAH C, MD
DIRECTOR
DEPARTMENT OF PEDIATRICS,
HEMATOLOGY AND ONCOLOGY
UNIVERSITY OF CALIFORNIA LOS ANGELES
LOS ANGELES, CA 90095

FREYER, DAVID R, DO
MEDICAL DIRECTOR
DEPARTMENT OF THE CHILDRENS CENTER FOR
CANCER AND BLOOD DISEASES
UNIVERSITY OF SOUTHERN CALIFORNIA- KECK SOM
LOS ANGELES, CA 90027-6016

FRIESE, CHRISTOPHER R, PHD, FAAN
PROFESSOR
DEPARTMENT OF NURSING
HEALTH MANAGEMENT AND POLICY
ROGEL CANCER CENTER
UNIVERSITY OF MICHIGAN
ANN ARBOR, MI 48109

GOLDSBY, ROBERT EDWARD, MD
PROFESSOR OF CLINICAL PEDIATRICS
DEPARMENT OF PEDIATRIC HEMATOLOGY/ONCOLOGY
BENIOFF CHILDRENS HOSPITAL
UNIVERSITY OF CALIFORNIA, SAN FRANCISO
SAN FRANCISCO, CA 94901

GRACIA, CLARISA R., MD
PROFESSOR
DEPARTMENT OF OBSTETRICS AND GYNECOLOGY
PENN FERTILITY CARE
UNIVERSITY OF PENNYSYLVANIA
PHILADELPHIA, PA 19104

GREER, JOSEPH ANDREW, PHD
ASSISTANT PROFESSOR
DEPARTMENT OF PSYCHOLOGY
HARVARD MEDICAL SCHOOL
MASSACHUSETTS GENERAL HOSPITAL CANCER CENTER
BOSTON, MA 02114

HAYES-LATTIN, BRANDON M, MD
PROFESSOR
DIVISION OF HEMATOLOGY AND MEDICAL ONCOLOGY
KNIGHT CANCER INSTITUTE
OREGON HEALTH AND SCIENCE UNIVERSITY
PORTLAND, OR 97239

KAZAK, ANNE E, PHD
PROFESSOR
DEPARTMENT FOR THE CENTER
FOR HEALTHCARE DELIVERY SCIENCE
NEMOURS CHILDREN'S HEALTH CARE SYSTEM
WILMINGTON, DE 19803

KEPKA, DEANNA LEE, PHD
ASSOCIATE PROFESSOR
DEPARTMENT OF NURSING
HUNTSMAN CANCER INSTITUTE
UNIVERSITY OF UTAH
SALT LAKE CITY, UT 84112

KUNIN-BATSON, ALICIA S, PHD
ASSISTANT PROFESSOR
DEPARTMENT OF PEDIATRICS
UNIVERSITY OF MINNESOTA MEDICAL SCHOOL
MINNEAPOLIS, MN 55414

LIU, LEI, PHD
PROFESSOR
DIVISION OF BIOSTATISTICS
WASHINGTON UNIVERSITY
ST. LOUIS, MO 63110

LOW, CARISSA A, PHD
ASSISTANT PROFESSOR
DEPARTMENT OF MOBILE SENSING AND
HEALTH INSTITUTE
UPMC HILLMAN CANCER CENTER
UNIVERSITY OF PITTSBURGH
PITTSBURGH, PA 15213

MANNE, SHARON L, PHD
PROFESSOR AND CHIEF
DEPARTMENT OF MEDICINE
CENTER OF EXCELLENCE IN
CANCER SURVIVORSHIP
ROBERT WOOD JOHNSON MEDICAL SCHOOL
NEW BRUNSWICK, NJ 08903

MEADE, CATHY D, PHD, FAAN
PROFESSOR
DEPARTMENT OF POPULATION SCIENCE
HEALTH OUTCOMES AND BEHAVIOR
MOFFITT CANCER CENTER
UNIVERSITY OF SOUTH FLORIDA
TAMPA, FL 33612

MOORE, JUSTIN BRIAN, PHD
ASSOCIATE PROFESSOR
DEPARTMENT OF FAMILY AND COMMUNITY MEDICINE,
EPIDEMIOLOGY & PREVENTION AND
IMPLEMENTATION SCIENCE
WAKE FOREST SCHOOL OF MEDICINE
WINSTON-SALEM, NC 27157

PERENTESIS, JOHN PETER, MD
PROFESSOR
DEPARTMENT OF PEDIATRICS
KLEISINGER CHAIR FOR NEW CANCER THERAPIES
CINCINNATI CHILDREN’S HOSPITAL
UNIVERSITY OF CINCINNATI COLLEGE OF MEDICINE
CINCINNATI, OH 45229-3039

PFEIFER, MARK P, MD
PROFESSOR
DEPARTMENT OF MEDICINE
UNIVERSITY OF LOUISVILLE HOSPITAL
LOUISVILLE, KY 40202

QUINN, GWENDOLYN P, CERTIFICAT, PHD
LIVIA WAN ENDOWED PROFESSOR
DEPARTMENT OF OBSTETRICS, GYNECOLOGY
AND POPULATION HEALTH
SCHOOL OF MEDICINE
NEW YORK UNIVERSITY
NEW YORK, NY 10016

RATAIN, MARK J, MD
PROFESSOR OF MEDICINE
DEPARTMENT OF MEDICINE
PRITZKER SCHOOL OF MEDICINE
UNIVERSITY OF CHICAGO
CHICAGO, IL 60637-1463

SALZ, TALYA, PHD
RESEARCH SCIENTIST
DEPARTMENT OF EPIDEMIOLOGY AND BIOSTATISTICS
MEMORIAL SLOAN-KETTERING CANCER CENTER
NEW YORK, NY 10065

SHERWOOD, PAULA R, PHD, FAAN
PROFESSOR
DEPARTMENT OF ACUTE AND TERTIERY CARE
UNIVERSITY OF PITTSBURGH
PITTSBURGH, PA 15261

SONG, LIXIN, PHD, FAAN
ASSOCIATE PROFESSOR
DIVISION OF CANCER PREVENTION AND CONTROL
SCHOOL OF NURSING
THE UNIVERSITY OF NORTH CAROLINA AT CHAPEL HILL
CHAPEL HILL, NC 27599

SPAULDING, AARON, MHA, PHD
ASSOCIATE CONSULTANT
DEPARTMENT OF HEALTH SCIENCES RESEARCH
DIVISION OF HEALTH CARE POLICY AND RESEARCH
MAYO CLINIC ROBERT D AND PATRICIA E KERN
JACKSONVILLE, FL 32224

STERN, MARILYN, PHD
PROFESSOR
DEPARTMENT OF CHILD AND FAMILY STUDIES
COLLEGE OF BEHAVIORAL AND COMMUNITY SCIENCES
MOFFITT CANCER CENTER
UNIVERSITY OF SOUTH FLORIDA
TAMPA, FL 33612

VANNATTA, KATHRYN, PHD
PROFESSOR
DEPARTMENT OF PSYCHOLOGY
CENTER FOR BIOBEHAVIORAL HEALTH
RESEARCH INSTITUTE
NATIONWIDE CHILDREN'S HOSPITAL
COLUMBUS, OH 43205

YANEZ, BETINA, PHD
ASSOCIATE PROFESSOR
DEPARTMENT OF PATIENT ENGAGEMENT
AND CANCER SURVIVORSHIP INSTITUTE
ROBERT H. LURIE COMPREHENSIVE CANCER CENTER
NORTHWESTERN UNIVERSITY
CHICAGO, IL 60611

YENNU, SRIRAM, MD
ASSOCIATE PROFESSOR
DEPARTMENT OF PALLIATIVE
DIVISION OF CANCER MEDICINE
REHABILITATION AND INTEGRATIVE MEDICINE
THE UNIVERSITY OF TEXAS MD ANDERSON CANCER CENTER
HOUSTON, TX 77030

MAIL REVIEWER(S)

BENTZEN, SOREN M, PHD, DSC
PROFESSOR
DEPARTMENT OF EPIDEMIOLOGY AND PUBLIC HEALTH
UNIVERSITY OF MARYLAND
BALTIMORE, MD 21201

LONG, JIRONG, PHD
ASSOCIATE PROFESSOR
VANDERBILT EPIDEMIOLOGY CENTER
VANDERBILT UNIVERSITY MEDICAL CENTER
NASHVILLE, TN 37203

MUELLER, SABINE, MD, PHD
ASSOCIATE PROFESSOR
DEPARTMENT OF NEUROLOGY
UNIVERSITY OF CALIFORNIA, SAN FRANCISCO
SAN FRANCISCO, CA 94143

SHAH, BIJAL DINESH, MD
SECTION LEADER
DEPARTMENT OF MALIGNANT HEMATOLOGY
MOFFITT CANCER CENTER
TAMPA, FL 33612

SCIENTIFIC REVIEW OFFICER

MEEKER, TIMOTHY C., MD, BS
SCIENTIFIC REVIEW OFFICER
SPECIAL REVIEW BRANCH
DIVISION OF EXTRAMURAL ACTIVITIES
NATIONAL CANCER INSTITUTE
NATIONAL INSTITUTES OF HEALTH
ROCKVILLE, MD 20850

EXTRAMURAL SUPPORT ASSISTANT

GRADINGTON-JONES, IMELA
PROGRAM SPECIALIST
SPECIAL REVIEW BRANCH
DIVISION OF EXTRAMURAL ACTIVITIES
NATIONAL CANCER INSTITUTE SHADY GROVE
NATIONAL INSTITUTES OF HEALTH
BETHESDA, MD 20892-9750

Consultants are required to absent themselves from the room during the review of any application if their presence would constitute or appear to constitute a conflict of interest.
